# Descriptive, Retrospective Study of the Clinical Characteristics of Asymptomatic COVID-19 Patients

**DOI:** 10.1128/mSphere.00922-20

**Published:** 2020-10-07

**Authors:** Huan Han, Zaichao Xu, Xiaoming Cheng, Youquan Zhong, Li Yuan, Fubing Wang, Yan Li, Fang Liu, Yingan Jiang, Chengliang Zhu, Yuchen Xia

**Affiliations:** a Department of Clinical Laboratory, Renmin Hospital of Wuhan University, Wuhan, China; b State Key Laboratory of Virology, School of Basic Medical Sciences, Wuhan University, Wuhan, China; c Hubei Province Key Laboratory of Allergy and Immunology, School of Basic Medical Sciences, Wuhan University, Wuhan, China; d Liver Diseases Branch, National Institute of Diabetes and Digestive and Kidney Diseases, National Institutes of Health, Bethesda, Maryland, USA; e Department of Laboratory Medicine, Zhongnan Hospital of Wuhan University, Wuhan, China; f Department of Pathogen Biology, School of Basic Medicine, Tongji Medical College, Huazhong University of Science and Technology, Wuhan, China; g State Key Laboratory of Virology, College of Life Sciences, Wuhan University, Wuhan, China; h Department of Infectious Diseases, Renmin Hospital, Wuhan University, Wuhan, China; National Institute of Allergy and Infectious Diseases

**Keywords:** COVID-19, asymptomatic, antibody, immune response, liver function

## Abstract

Since the outbreak of coronavirus disease 2019 (COVID-19) in Wuhan, China, it has rapidly spread around the world. Persons with asymptomatic disease exhibit viral shedding, resulting in transmission, which presents disease control challenges. However, the clinical characteristics of these asymptomatic individuals remain elusive. We collected samples of 25 asymptomatic and 27 symptomatic COVID-19 patients. Viral titers of throat swabs were determined by quantitative reverse transcription-PCR (qRT-PCR). COVID-19 IgG and IgM were examined. Complete blood counts were determined, and serum biochemistry panels were performed. Cytokines, including gamma interferon (IFN-γ), tumor necrosis factor alpha (TNF-α), interleukin 2 (IL-2), IL-4, IL-6, and IL-10 were evaluated. T cell, B cell, and NK cell counts were measured using flow cytometry. Although similar viral loads were detected, asymptomatic patients had significantly faster virus turnover than symptomatic patients. Additionally, asymptomatic patients had higher counts of lymphocytes, T cells, B cells, and NK cells. While liver damage was observed in symptomatic patients, as indicated by elevated liver enzymes and decreased liver-synthesized proteins in the blood, asymptomatic patients showed normal liver measurements. Lactate dehydrogenase, a COVID-19 risk factor, was significantly lower in asymptomatic patients. These results suggest that asymptomatic COVID-19 patients had normal clinical indicators and faster viral clearance than symptomatic patients. Lymphocytes may play a role in their asymptomatic phenotype. Since asymptomatic patients may be a greater risk of virus transmission than symptomatic patients, public health interventions and a broader range of testing may be necessary for the control of COVID-19.

**IMPORTANCE** Asymptomatic transmission of severe acute respiratory syndrome coronavirus 2 (SARS-CoV-2) is a potential problem for pandemic control through public health strategies. Our results demonstrate that asymptomatic COVID-19 patients have better outcomes than symptomatic patients. This may have been due to more active cellular immune responses and normal liver function. Since asymptomatic patients have no clinical symptoms which can easily prevent timely diagnosis and treatment, they may cause a greater risk of virus transmission than symptomatic patients, which poses a major challenge to infection control. Evidence suggests that nonpharmaceutical public health interventions, like social distancing and face mask ordinances, play important roles in the control of COVID-19. Looking forward, it may be necessary to proceed cautiously while reopening businesses in areas of epidemicity to prevent potential waves of COVID-19 in the future.

## Abstract (precis)

Asymptomatic transmission of severe acute respiratory syndrome coronavirus 2 (SARS-CoV-2) is a potential problem for pandemic control through public health strategies. Our results demonstrate that asymptomatic COVID-19 patients have better outcomes than symptomatic patients. This may have been due to more active cellular immune responses and normal liver function. Since asymptomatic patients have no clinical symptoms which can easily prevent timely diagnosis and treatment, they may cause a greater risk of virus transmission than symptomatic patients, which poses a major challenge to infection control. Evidence suggests that nonpharmaceutical public health interventions, like social distancing and face mask ordinances, play important roles in the control of COVID-19. Looking forward, it may be necessary to proceed cautiously while reopening businesses in areas of epidemicity to prevent potential waves of COVID-19 in the future.

## INTRODUCTION

In December 2019, a number of cases of pneumonia with an unknown cause were reported in Wuhan, China (1). Subsequently, the pathogen of this disease was identified as novel severe acute respiratory syndrome coronavirus 2 (SARS­CoV­2) (2) and the disease was named coronavirus disease 2019 (COVID-19) by the World Health Organization (WHO). As of 30 September 2020, COVID-19 has affected more than 200 countries, with 33,561,077 confirmed cases and 1,005,004 confirmed deaths worldwide (3). As no vaccine or effective treatments are currently available, COVID-19 continues to spread across the world and poses a great health burden to many countries.

One of the principal challenges in disease control of COVID-19 lies in the recognition that infected but asymptomatic patients can still shed infectious virus. Due to the lack of symptoms, this group of patients is easily overlooked by screening measures which would otherwise result in self-quarantine. Limited study of those patients has found that the incubation period of asymptomatic infection may be as long as 29 days, and human-to-human transmission can occur during this period (4–6). This is due to asymptomatic carriers harboring similar levels of SARS-CoV-2 based on nucleic acid reverse transcription-PCR (RT-PCR) testing (7–9). Thus far, many studies have analyzed the clinical characteristics of SARS-CoV-2-infected patients presenting levels of illness ranging from mild to severely critical (10, 11). However, detailed clinical profiles of those asymptomatic individuals are not well documented. In this study, we enrolled 25 asymptomatic and 27 symptomatic COVID-19 patients and performed systematic analysis of different clinical characteristics. Our results reveal the pathogenesis of asymptomatic SARS-CoV-2 infection and provide important information for its clinical management.

## RESULTS

**FIG 1:**
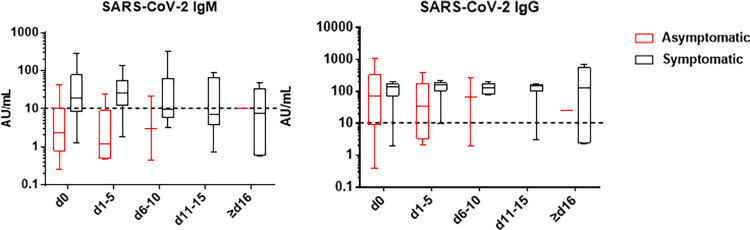
Dynamic changes of anti-SARS-CoV-2 IgM and IgG antibodies in asymptomatic and symptomatic patients. The box plots display anti-SARS-CoV-2 IgM and IgG concentrations at the 25th, 50th, and 75th percentiles. The cutoff value was defined as 10 AU/ml (dotted line) according to the manufacturer’s instructions by using a SARS-CoV-2 IgM and IgG antibody chemiluminescence detection kit. The *x* axis represents the patient's hospital day. d0 represents the day of hospital admission.

We studied a total of 52 individuals whose clinical measurements are available in Renmin Hospital of Wuhan University in Wuhan, China. Based on their clinical presentation during the course before viral clearance, patients were divided into symptomatic and asymptomatic groups. All patients recovered (undetectable SARS-CoV-2) by the time of discharge, and the clinical and laboratory characteristics of the patients are summarized in Table 1. Asymptomatic and symptomatic COVID-19 patient groups had comparable ages, genders, and comorbidities. However, asymptomatic patients had significantly faster recovery than symptomatic patients, as shown by the median numbers of days of hospitalization (9 days versus 26 days; *P* < 0.001). As there was no difference in viral loads between the two groups, these data indicate that asymptomatic patients clear the virus faster. While COVID-19 IgG quantifications indicated similar results for the two groups, IgM levels were significantly lower in the asymptomatic group. Dynamic data demonstrated that all the patients had relatively stable IgG levels during hospitalization regardless of whether they were symptomatic or not, and the IgM level of the symptomatic group slowly decreased over time (Fig. 1). These results suggest that asymptomatic patients may have been exposed to the virus at a much earlier time point or that they may have compromised IgM production.

**TABLE 1:** The clinical characteristics and laboratory examination results of patients with COVID-19^*a*^

Characteristic	Specific aspect	Result for indicated group		*P* value
		Asymptomatic	Symptomatic	
		*n* = 25	*n* = 27	
Mean age ± SD (yr)		47.1 ± 19.8	50.1 ± 13.8	0.528
Gender, no. (%) of patients	Male	12 (48.00)	15 (55.56)	0.586
	Female	13 (52.00)	12 (44.44)	
Comorbidity, no. (%) of patients	Any	8 (32.00)	9 (33.33)	0.918
	Hypertension	3 (12.00)	4 (14.81)	1.000
	Diabetes	2 (8.00)	2 (7.41)	1.000
	Cardiovascular diseases	1 (4.00)	1 (3.70)	1.000
	Hepatitis or fatty liver	2 (8.00)	1 (3.70)	0.945
	Chronic bronchitis, bronchial asthma	1 (4.00)	1 (3.70)	1.000
	Pharyngitis	0	1 (3.70)	1.000
	Hyperlipidemia	0	1 (3.70)	1.000
	Intrahepatic cholangiocarcinoma	1 (4.00)	0	0.481
	Gastritis	1 (4.00)	0	0.481
	Rheumatoid arthritis	1 (4.00)	0	0.481
	Alzheimer's disease	2 (8.00)	0	0.226
COVID-19 treatments, no. (%) of patients	Chloroquine	2 (8.00)	2 (7.41)	1.000
	Arbidol	10 (40.00)	14 (51.85)	0.392
	Traditional Chinese medicine	11 (44.00)	14 (51.85)	0.571
	Others (oseltamivir, ribavirin, or interferon)	14 (56.00)	17 (62.96)	0.609
No. of days of hospitalization^*b*^		9 (7, 13)	26 (19, 36)	**<0.001**
				
Laboratory results				
		*n* = 5	*n* = 21	
SARS-CoV-2 RNA	Novel CoV ORF1ab (*C_T_*)	37.74 ± 2.96	36.47 ± 3.79	0.496
				
		*n* = 12	*n* = 17	
SARS-CoV-2 RNA	Novel CoV NP (*C_T_*)	36.24 ± 1.86	37.48 ± 4.17	0.344
				
SARS-CoV-2 antibodies^*b*^		*n* = 23	*n* = 27	
	IgG novel CoV (AU/ml)	70.70 (8.95, 348.10)	138.78 (72.55, 166.45)	0.514
	IgM novel CoV (AU/ml)	2.31 (0.82, 10.36)	18.42 (8.62, 75.90)	**<0.001**
				
Blood routine^*b*^		*n* = 25	*n* = 27	
	WBC (×10^9^/liter)	6.74 (5.32, 8.02)	5.72 (4.45, 7.56)	0.230
	Neu (×10^9^/liter)	3.78 (2.88, 5.49)	3.41 (2.71, 5.56)	0.812
	LYM (×10^9^/liter)	1.87 ± 0.92	1.43 ± 0.60	**0.046**
	Mono (×10^9^/liter)	0.53 ± 0.21	0.51 ± 0.19	0.663
	EOS (×10^9^/liter)	0.09 (0.04, 0.18)	0.03 (0.02, 0.11)	**0.039**
	BASO (×10^9^/liter)	0.03 (0.02, 0.05)	0.02 (0.01, 0.03)	**0.016**
	RBC (×10^12^/liter)	4.38 ± 0.89	**4.09 ± 0.55↓**	0.169
	Hb (g/liter)	131.40 ± 22.92	**128.78 ± 15.31↓**	0.633
	HCT (liter/liter)	**0.38 ± 0.06↓**	**0.37 ± 0.04↓**	0.355
	MCV (fl)	88.70 (86.35, 92.45)	89.70 (87.80, 92.60)	0.296
	MCH (pg)	30.80 (29.80, 31.65)	31.70 (30.00, 32.40)	0.169
	MCHC (g/liter)	342.00 (333.00, 349.00)	346.00 (337.00, 353.00)	0.244
	RDW-SD (fl)	41.40 (39.65, 44.45)	39.90 (37.20, 44.50)	0.197
	RDW-CV	0.13 (0.12, 0.14)	0.12 (0.12, 0.13)	0.011
	PLT (×10^9^/liter)	226.32 ± 73.09	259.44 ± 97.66	0.175
	PCT (%)	0.002 (0.002, 0.003)	0.003 (0.002, 0.003)	0.072
	MPV (fl)	10.24 ± 1.16	10.54 ± 1.28	0.379
	PDW (fl)	10.50 (10.25, 13.00)	11.50 (9.90, 13.40)	0.640
	P-LCR (%)	26.39 ± 9.40	28.89 ± 10.21	0.365
				
Cellular immune response		*n* = 21	*n* = 27	
	CD3^+^ (/μl)	1,286.00 ± 584.61	905.41 ± 427.72	**0.016**
	CD4^+^ (/μl)	760.52 ± 363.08	525.22 ± 282.92	**0.015**
	CD8^+^ (/μl)	464.43 ± 249.83	348.70 ± 216.75	0.093
	CD19^+^ (/μl)	272.57 ± 215.65	190.70 ± 83.89	0.113
	CD16^+^ CD56^+^ (/μl)	197.52 ± 89.96	161.41 ± 97.12	0.194
Cytokines^*b*^		*n* = 15	*n* = 24	
	IL-2 (pg/ml)	3.58 (3.49, 3.95)	3.75 (3.18, 4.26)	0.679
	IL-4 (pg/ml)	3.15 (2.88, 3.56)	3.36 (3.00, 4.28)	0.202
	IL-6 (pg/ml)	5.77 (4.36, 11.05)	10.45 (5.25, 17.58)	0.110
	IL-10 (pg/ml)	5.98 (5.45, 6.78)	5.09 (4.52, 6.54)	0.097
	TNF-α (pg/ml)	3.65 (3.12, 4.28)	3.40 (3.02, 5.18)	0.898
	IFN-γ (pg/ml)	3.21 (3.17, 3.44)	3.73 (2.94, 4.27)	0.484
				
Serum biochemistry^*b*^		*n* = 24	*n* = 27	
	ALT (U/liter)	21.13 ± 13.25	32.26 ± 18.24	**0.017**
	AST (U/liter)	17.00 (15.00, 22.50)	26.00 (21.00, 33.00)	**0.001**
	ALP (U/liter)	68.00 (56.05, 97.50)	68.00 (52.00, 79.00)	0.412
	GGT (U/liter)	18.50 (13.00, 46.75)	34.00 (16.00, 53.00)	0.213
	TP (g/liter)	66.26 ± 5.92	**62.57 ± 5.23↓**	**0.022**
	ALB (g/liter)	42.27 ± 5.42	**38.50 ± 4.15↓**	**0.007**
	GLB (g/liter)	23.32 (21.75, 25.28)	23.20 (21.00, 25.50)	0.955
	TBIL (μmol/liter)	12.15 (10.73, 17.93)	10.50 (8.20, 13.80)	0.086
	DBIL (μmol/liter)	4.00 (3.08, 5.00)	3.50 (2.40, 4.40)	0.312
	Urea (mmol/liter)	5.32 ± 1.92	4.57 ± 1.90	0.172
	Cr (μmol/liter)	62.38 ± 14.68	59.26 ± 12.19	0.412
	TCO2 (mmol/liter)	26.34 ± 2.40	26.00 ± 2.58	0.632
	UA (μmol/liter)	341.58 ± 91.41	266.44 ± 83.94	**0.004**
	Glu (mmol/liter)	5.01 (4.20, 6.07)	5.02 (4.47, 6.66)	0.699
	K (mmol/liter)	3.91 ± 0.40	4.06 ± 0.40	0.193
	Na (mmol/liter)	142.65 ± 3.25	141.65 ± 3.71	0.311
	Cl (mmol/liter)	106.20 (104.08, 107.95)	106.40 (104.80, 107.60)	0.699
	Ca (mmol/liter)	2.31 ± 0.13	2.18 ± 0.11	**<0.001**
	Mg (mmol/liter)	0.84 ± 0.07	0.87 ± 0.08	0.135
	IP (mmol/liter)	1.33 ± 0.28	1.23 ± 0.18	0.145
	OSMO (mosmol/liter)	286.83 ± 6.84	285.99 ± 9.74	0.727
	TCh (mmol/liter)	4.30 ± 0.96	4.04 ± 0.99	0.359
	TG (mmol/liter)	0.96 (0.85, 1.66)	1.53 (0.91, 1.96)	0.151
	HDL-Ch (mmol/liter)	1.24 (0.95, 1.45)	**0.85 (0.76, 1.18)↓**	**0.002**
	LDL-Ch (mmol/liter)	2.53 ± 0.87	2.55 ± 0.80	0.913
	Lp(a) (mg/liter)	136.00 (74.50, 234.75)	95.00 (52.00, 235.00)	0.748
	CK (U/liter)	55.50 (37.75, 71.00)	58.00 (45.00, 78.00)	0.497
	LDH (U/liter)	177.00 (145.00, 206.25)	220.00 (187.00, 283.00)	**0.003**
	eGFR (ml/min)	106.78 ± 19.01	107.93 ± 10.22	0.786

a **↓** means below the normal range; **↑** means above the normal range. Boldface indicates significance. The data indicate the time since the admission of the first plasma sample of patients taken for clinical testing in this study. ALB, albumin; ALP, alkaline phosphatase; ALT, alanine aminotransferase; AST, aspartate aminotransferase; AU, arbitrary units; BASO, basophil count; CK, creatine kinase; COVID-19, coronavirus disease 2019; Cr, creatinine; *C_T_*, threshold cycle; CV, coefficient of variation; DBIL, direct bilirubin; eGFR, epidermal growth factor receptor; EOS, eosinophil count; GGT, gamma-glutamyl transpeptidase; GLB, globulin; Glu, glucose; Hb, hemoglobin; HCT, hematocrit; HDL-Ch, high-density lipoprotein-cholesterol; IP, phosphorus; LDH, lactate dehydrogenase; LDL-Ch, low-density lipoprotein-cholesterol; Lp(a), lipoprotein (a); LYM, lymphocyte count; MCH, mean corpuscular hemoglobin; MCHC, mean corpuscular hemoglobin concentration; MCV, mean corpuscular volume; Mono, monocyte count; MPV, mean platelet volume; Neu, neutrophil count; NP, nucleocapsid protein; OSMO, osmotic pressure; PCT, plateletcrit; PDW, platelet distribution width; P-LCR, platelet-large cell ratio; PLT, platelet; RBC, red blood cell; RDW-CV, red blood cell distribution width coefficient of variation; RDW-SD, red blood cell distribution width standard deviation; SARS-CoV-2, severe acute respiratory syndrome coronavirus 2; TBIL, total bilirubin; TCh, total cholesterol; TCO2, total carbon dioxide; TG, triglyceride; TP, total protein; UA, uric acid; WBC, white blood cell.

b Numbers in parentheses are coefficients of variation.

**FIG 2:**
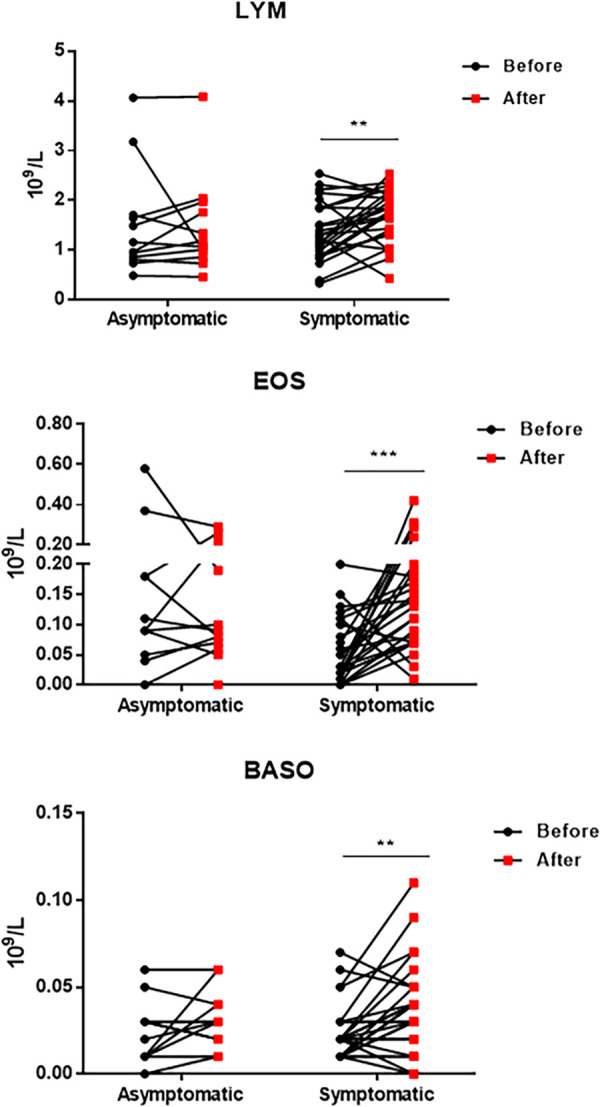
Comparison of lymphocyte counts of symptomatic and asymptomatic COVID-19 patients before and after treatments. Lymphocyte, basophil, and eosinophil counts from symptomatic and asymptomatic patients before and after COVID-19 treatments have been determined. LYM, lymphocytes; BASO, basophils; EOS, eosinophils. Wilcoxon's signed-rank test was used. *, *P* < 0.05; **, *P* < 0.01; ***, *P* < 0.001.

**FIG 3:**
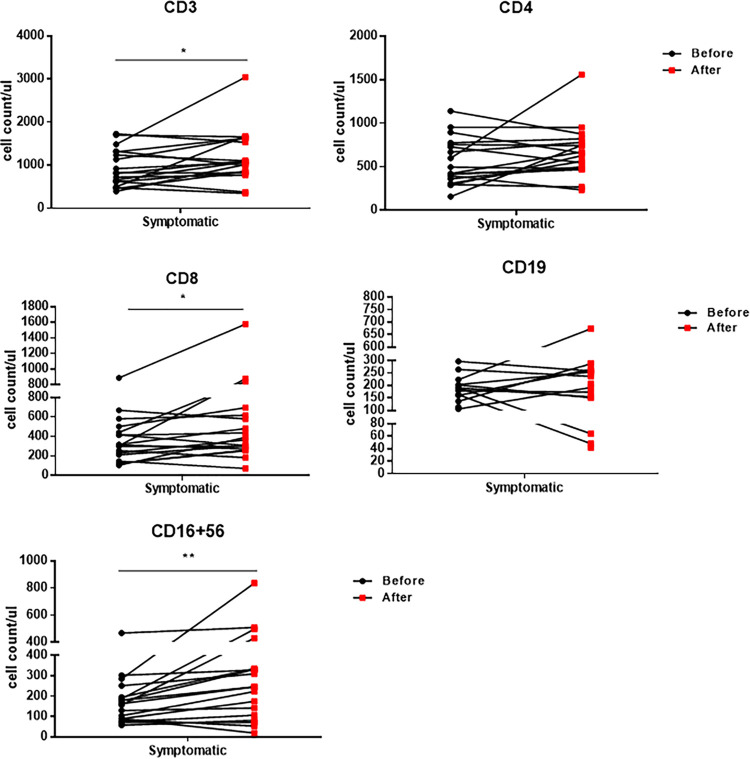
Changes in the cellular immune responses of symptomatic COVID-19 patients before and after treatments. CD3 total T cell, CD4 T cell, CD8 T cell, CD19 B cell, and CD16^+^ CD56^+^ NK cell counts from symptomatic patients before and after COVID-19 treatments were analyzed. Wilcoxon's signed-rank test was used. *, *P* < 0.05; **, *P* < 0.01; ***, *P* < 0.001.

Complete blood counts revealed that asymptomatic patients had higher counts of lymphocytes, eosinophils, and basophils than symptomatic patients. Analyzed by flow cytometry, total CD3^+^ T cell, CD4^+^ T cell, CD19^+^ B cell, and CD16^+^ CD56^+^ NK cell counts were higher in asymptomatic patients. Among them, the levels of T cells, specifically CD4^+^ T cells, were significantly statistically different. To further investigate the role of these immune cells, we compared their counts before and after treatments in all patients. As shown in Fig. 2 and Supplemental Table S1 after clearance of SARS-CoV-2, the counts of lymphocytes, basophils, and eosinophils in symptomatic patients were restored. Since most asymptomatic patients had only one measurement of their cellular immune response, we present only the change in these markers in symptomatic patients (Table S1 and Fig. 3). Interestingly, total T cell, CD8^+^ T cell, and NK cell counts after viral clearance were significantly increased. Additionally, the longitudinal changes of lymphocytes (Fig. S1) and immune cells (Fig. S2) of symptomatic COVID-19 patients were analyzed. In keeping with previous findings, basophils and eosinophils as well as immune cells were slightly increased during hospitalization. Together, these results highlight the role of immune cells in controlling SARS-CoV-2 in infected patients.

10.1128/mSphere.00922-20.1 FIG S1 Kinetics of lymphocytes in symptomatic COVID-19 patients during hospitalization. The counts of lymphocytes, eosinophils, and basophils in symptomatic COVID-19 patients during hospitalization are presented. The *x* axis represents days after admission. Medians with ranges are presented. Download FIG S1, PDF file, 0.03 MB. Copyright © 2020 Han et al. 2020 Han et al. This content is distributed under the terms of the Creative Commons Attribution 4.0 International license.

10.1128/mSphere.00922-20.2 FIG S2 Kinetics of immune cells in symptomatic COVID-19 patients during hospitalization. The counts of different immune cells in symptomatic COVID-19 patients during hospitalization are presented. The *x* axis represents days after admission. Medians with ranges are presented. Download FIG S2, PDF file, 0.05 MB. Copyright © 2020 Han et al. 2020 Han et al. This content is distributed under the terms of the Creative Commons Attribution 4.0 International license.

10.1128/mSphere.00922-20.4 TABLE S1 Selected markers of symptomatic and asymptomatic COVID-19 patients before and after treatments. Download Table S1, PDF file, 0.1 MB. Copyright © 2020 Han et al. 2020 Han et al. This content is distributed under the terms of the Creative Commons Attribution 4.0 International license.

We previously reported that elevated inflammatory cytokine expression in COVID-19 patients and higher levels of cytokine storm are associated with more severe disease development (12). Herein, we also compare the levels of several representative cytokines that are known to be elevated in COVID-19 patients, including gamma interferon (IFN-γ), tumor necrosis factor alpha (TNF-α), interleukin 2 (IL-2), IL-4, IL-6, and IL-10 (12). However, no difference in inflammatory cytokine levels was observed between asymptomatic and symptomatic COVID-19 patients. Of note, all symptomatic COVID-19 patients in this study had only mild or moderate symptoms. Our results demonstrate that although some cytokine markers can be used to predict disease severity among symptomatic patients, they might lose predictive power when asymptomatic patients are included.

It has frequently been observed that besides injury to the lungs, damage to several other organs/cell types of infected patients occurs during the course of illness (13, 14). Increased alanine aminotransferase (ALT), aspartate aminotransferase (AST), and lactate dehydrogenase (LDH) were frequently found. We next checked serum biochemistry markers. Although the liver enzymes ALT and AST in both groups were within normal ranges, symptomatic patients had significantly higher levels. Along the same line, levels of albumin (ALB) and total protein (TP), two additional markers reflective of liver function, were significantly lower and below the normal range in symptomatic patients. In addition, lipid metabolism, which is carried out mainly in the liver, was also degraded in symptomatic patients, as demonstrated by lower levels of high-density lipoprotein. These results suggest that asymptomatic patients, unlike symptomatic patients, tend to have a lower risk of developing liver damage. Previous studies have proposed LDH (15) and creatine kinase (CK) (11) as risk factors for severe COVID-19. In our cohorts, asymptomatic patients had lower levels of LDH (Table 1). Interestingly, after COVID-19 treatments, AST and LDH declined in symptomatic patients (Fig. S3). These results suggest that asymptomatic patients have a lower risk of disease deterioration.

10.1128/mSphere.00922-20.3 FIG S3 Comparison of selected markers of symptomatic and asymptomatic COVID-19 patients before and after treatments. Selected liver function markers and COVID-19 severity indicators from symptomatic and asymptomatic patients before and after COVID-19 treatments have been determined. ALB, albumin; ALT, alanine aminotransferase; AST, aspartate aminotransferase; TP, total protein; LDH, lactate dehydrogenase. The Wilcoxon signed-rank test was used. *, *P* < 0.05; **, *P* < 0.01; ***, *P* < 0.001. Download FIG S3, PDF file, 0.1 MB. Copyright © 2020 Han et al. 2020 Han et al. This content is distributed under the terms of the Creative Commons Attribution 4.0 International license.

## DISCUSSION

Given the high burden of COVID-19 worldwide, how SARS-CoV-2 infection directs a portion of patients to develop no symptoms needs to be evaluated. In this study, we systematically compared different complete blood counts, serum biochemistries, and immunologic responses from SARS-CoV-2-infected asymptomatic and symptomatic individuals. It was found that both groups had similar viral loads; however, asymptomatic patients had significantly decreased hospital usage and lower IgM than symptomatic patients. Additionally, asymptomatic patients had higher counts of lymphocytes, T cells, B cells, and NK cells. Impaired liver function was observed in symptomatic patients but not in asymptomatic patients. LDH, a crucial biomarker for the patient mortality rate, was significantly lower in asymptomatic patients. Our results suggest that asymptomatic COVID-19 patients have better outcomes than symptomatic patients, which may be due to a more active cellular immune response and normal liver function. It is exceedingly problematic for asymptomatic patients to be diagnosed and treated in a timely manner due to the nature of their disease presentation; thus, they may present a greater risk for virus transmission than symptomatic patients, which poses a major threat to infection control.

Several studies suggested a positive association between viral dose and the severity of COVID-19 (16, 17). However, the evidence of this correlation is limited by the retrospective nature of the studies, small sample sizes, and potential problems with selection bias. As in previous reports (18), the viral load in asymptomatic patients was similar to that found in symptomatic patients at the time of hospital admission. Care should be taken with the interpretation of the presence of viral RNA in specimens, as it does not always correlate with viral transmissibility because the virus may not remain intact. In this study, we measured solely viral loads using throat swabs. However, the duration of SARS-CoV-2 is significantly longer in stool samples than in respiratory and serum samples (17). As SARS-CoV-2 infects not only the respiratory system but also many other organs (19), it is worthwhile to evaluate other samples, like stool.

The interaction of SARS-CoV-2 and the immune system might explain why some COVID-19 patients were asymptomatic after virus infection. It was reported that over 80% of COVID-19 patients had lymphopenia (20), which is related to the severity of the disease (10). It is known that children appear to have much lower rates of symptomatic infection than adults (21), and less than 10% of infected children presented with lymphopenia (22). In this study, although lymphocyte counts of both groups were in the normal range, asymptomatic patients had significantly higher counts than symptomatic patients. In COVID-19 patients, the numbers of CD4^+^ and CD8^+^ T cells decreased, while the levels of IL-6 and IL-10 increased in severe cases (20). Further work is necessary to determine if these asymptomatic patients had past exposure to other coronaviruses which had somehow primed T cells to recognize and control SARS-CoV-2 upon infection. Recent reports provide some evidence of a cross-reactive CD4^+^ and CD8^+^ T cell response in patients with COVID-19 hypothesized to be due to exposure to other coronaviruses, including those from patients who have never been exposed to the SARS, Middle East respiratory syndrome (MERS), or COVID-19 coronaviruses (23). It has also been shown that the CD4^+^ and CD8^+^ T cell response is cross-reactive between the N proteins of both SARS and COVID-19 patients, with immunity to COVID-19 remaining for patients originally exposed to SARS-CoV (24). It has been further demonstrated that the memory CD4^+^ T cell population, which reacts to SARS-CoV-2 epitopes, can cross-react with similar sequences from common cold coronaviruses, with epitope homology of over 67% being associated with cross-reactivity for a majority of cases (25). This cross-reactive T cell response may influence asymptomatic patient susceptibility to COVID-19 disease and prevent them from developing severe symptoms, as asymptomatic patients exhibited a statistically significantly increased number of CD4^+^ T cells compared to those of symptomatic patients.

A recent study demonstrated that asymptomatic COVID-19 patients exhibited lower levels of several pro- and anti-inflammatory cytokines, and they speculated that asymptomatic individuals had a weaker immune response to SARS-CoV-2 infection (26). However, in our study, we did not observe significant differences in the cytokine responses in the two groups. The discrepancy between these two studies may have been caused by the different selection criteria of the symptomatic control group. To better compare the clinical characters of asymptomatic and symptomatic COVID-19 patients, we randomly selected symptomatic control patients from hospitalized mild- or moderate-COVID-19 patients to match the ages, genders, and comorbidities of the asymptomatic group. As we and others reported previously, several cytokines are associated with COVID-19 disease severity (12). This result suggested that asymptomatic patients have cytokine levels similar to those of patients with mild or moderate symptoms.

Liver damage caused by SARS-CoV-2 infection might present clinical challenges. In keeping with some published studies, our results showed that some COVID-19 patients have impaired liver function (27). Similar observations were reported with SARS and MERS patients (28). Although it was proposed that liver damage in COVID-19 patients may be due to drug hepatotoxicity or immune-mediated inflammation, the possibility of SARS-CoV-2 infection of liver cells cannot be excluded. By using single-cell transcriptome sequencing (RNA-seq), a recent study revealed significant enrichment of ACE2 expression in a major portion of the cholangiocytes and low expression of ACE2 in hepatocytes (29). Therefore, COVID-19 might bind to the target cell expressing cellular receptors which contribute to mediation of viral entry and impact liver function. Further studies on the detailed mechanisms of the viral life cycle and potential clinical manifestations and interventions should be conducted.

There are some limitations of the current study. First, the sample size is small. Second, compared with those of the symptomatic group, the results of dynamic immunologic changes in asymptomatic patients are incomplete. Third, although we have analyzed and compared the immune responses of the two groups, the detailed mechanisms of the immune dysregulation were not addressed. Future studies are needed to increase our knowledge of the pathogenesis of COVID-19 and provide a basis for disease control and new therapeutic strategies.

Although both COVID-19 and SARS are caused by coronaviruses, the differences of these two diseases are clear. Within 8 months, SARS was controlled after the virus had infected approximately 8,100 persons in limited geographic areas (30). However, within 6 months, SARS-CoV-2 infected more than 6 million people and continues to spread worldwide (3, 30). It is crucial to evaluate the burden of asymptomatic COVID-19 patients. COVID-19 transmission in the absence of symptoms reinforces the value of measures that prevent the spread of SARS-CoV-2 by infected persons who may not exhibit illness despite being infectious. Nonpharmaceutical public health interventions, like social distancing and face mask ordinances, together with virus screening play important roles in the control of COVID-19. Currently, widely used symptom-based screening alone misses a high proportion of infectious cases and was not enough to control transmission. The city of Wuhan, China, where the COVID-19 outbreak was first reported, recently launched a campaign to test its 11 million residents for SARS-CoV-2. Although it is time-intensive and costly, a “pooled testing” strategy to screen many residents at once might be considered and has been proposed. As activity resumes in areas of epidemicity, caution must be taken to prevent potential future waves of COVID-19.

## MATERIALS AND METHODS

### Patients.

Fifty-two confirmed COVID-19 patients admitted to Renmin Hospital of Wuhan University from 31 January 2020 to 16 April 2020, consisting of 25 asymptomatic and 27 symptomatic patients, were enrolled in this study. All asymptomatic patients were diagnosed during physical examination or presurgical testing and admitted or transferred to the special COVID-19 department immediately after confirmed diagnosis of infection. Symptomatic patients were randomly selected from hospitalized mild- or moderate-COVID-19 patients to match the ages, genders, and comorbidities of the asymptomatic group.

According to the guidelines in the *Diagnosis and Treatment for Novel Coronavirus Pneumonia* of the National Health Commission of China (seventh edition), all COVID-19 cases were confirmed according to positive respiratory RT-PCR tests (31). Confirmed cases were defined as positive (i) by RT-PCR detection of SARS-CoV-2 nucleic acid, (ii) when viral-genome sequencing results were highly homologous to those for SARS-CoV-2, (iii) when tests for serum SARS-CoV-2-specific IgM antibodies and IgG antibody reflected a conversion from negative to positive, or (iv) when the recovery period was 4 times or more that of the acute period. Asymptomatic cases were defined as a confirmed case with a positive SARS-CoV-2 nucleic acid RT-PCR test but without any symptoms of COVID-19, such as fever, gastrointestinal, or respiratory symptoms (7). The discharge criteria of the recovered patients include the following: the patient’s temperature had returned to normal for more than 3 days and the patient exhibited significantly improved respiratory symptoms, significant absorption of pulmonary lesions in chest computed tomography (CT) imaging, and at least two consecutive negative RNA test results separated by at least 24 h. All basal-line samples were collected immediately after hospitalization. The study was approved by the ethics committee of Renmin Hospital (file number WDRY2020-K066).

### SARS-CoV-2 RNA detection.

Real-time RT-PCR amplification of SARS-CoV-2 open reading frame 1ab (ORF1ab) and nucleocapsid protein (NP) gene fragments was performed on throat swabs as described previously (1).

### SARS-CoV-2 IgM and IgG assay.

Serum SARS-CoV-2 IgM and IgG antibodies were detected by using a SARS-CoV-2 IgM and IgG antibody chemiluminescence detection kit (catalog no. C86095M; YHLO Biotech, Shenzhen, China) on an iFlash3000 automatic chemiluminescence immunoassay analyzer (YHLO Biotech, Shenzhen, China) according to the manufacturer’s instructions. The kit contained two recombinant SARS-CoV-2 antigens for nucleoprotein and spike protein. The sensitivity and specificity for IgM are 88.2% and 99.0%, respectively, and for IgG are 97.8% and 97.9%, respectively. The cutoff for both the IgM and the IgG test was 10.0 arbitrary units (AU)/ml.

### Complete blood count.

Venous blood was collected in an anticoagulation tube from the patients in a fasting state. The blood specimens were then placed at a suitable temperature. Before the routine blood examination was performed, the blood samples were mixed repeatedly and analyzed by a multifunctional automatic hematology analyzer (XN9000; Sysmex, Kobe, Japan).

### Serum biochemistry panel.

The venous blood of the patients was obtained using the methods described for routine blood analysis. Multiple biochemical indicators were evaluated using an automatic serum biochemical analyzer (ADVIA 2400; Siemens, Munich, Germany).

### Cytokine test.

Approximately 3 to 5 ml of peripheral blood was obtained from each patient, and the serum samples were separated by centrifugation at 2,000 rpm for 20 min. Serum cytokines were tested using the BD FACSCalibur flow cytometer (San Jose, CA, USA) and a human Th1/Th2 cytokine kit (Ceger, Hangzhou, China) according to the manufactures’ instructions. Briefly, 25 μl of serum sample was mixed with capture antibody-coupled beads and then with 25 μl of fluorescently labeled detection antibodies. The samples were mixed and incubated at room temperature in the dark. After incubation for 2.5 h, beads were washed and resuspended with phosphate-buffered saline (PBS). A recombinant protein standard of each cytokine was included to serve as an internal control. Detection was performed by flow cytometry.

### Cellular immunity test.

The BD Multitest IMK kit (San Jose, CA, USA) was used to determine T cell, NK cell, and B cell counts. Briefly, 50 μl of whole blood from each patient was added to 10 μl of CD3, CD8, CD4, CD16^+^ CD56^+^, and CD19 magnetic beads, which were incubated for 15 min in the dark, after which 450 μl of a hemolytic agent was added. After 10 min of incubation, the samples were measured by using a BD FACSCalibur flow cytometer (San Jose, CA, USA).

### Statistical analysis.

All statistical analysis was performed with SPSS software version 22.0, and *P* values of less than 0.05 were considered statistically significant. Continuous variables were evaluated using the median and interquartile range (IQR) values or expressed as means ± standard deviations (SD). Categorical variables were expressed as counts and percentages in each category. Wilcoxon rank sum tests or *t* tests were applied to continuous variables. Chi-square tests were used for categorical variables.
