# Supplementary figures and images for: Descriptive, Retrospective Study of the Clinical Characteristics of Asymptomatic COVID-19 Patients
Source: mSphere. 2020 Oct 7;5(5):e00922-20. doi: 10.1128/mSphere.00922-20 (PMC7568656; doi:10.1128/mSphere.00922-20)

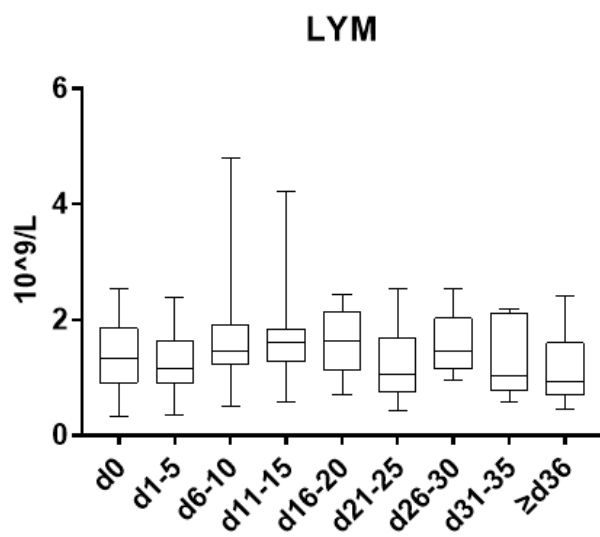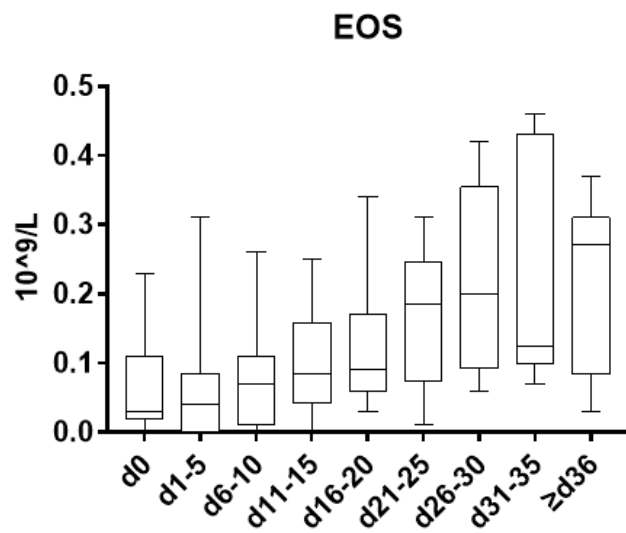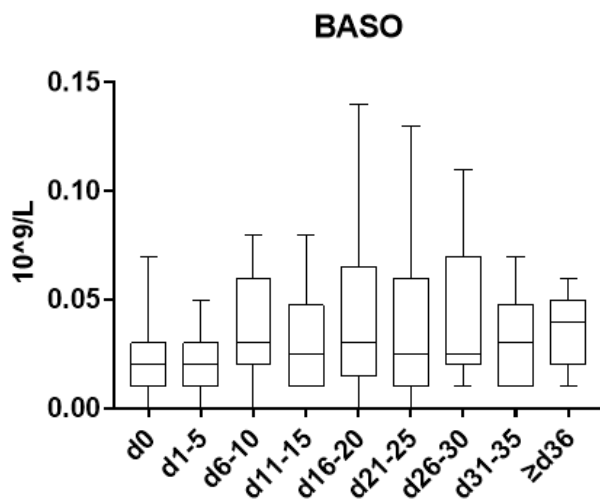

Supplement: FIG S1 [file mSphere.00922-20-sf001.pdf]

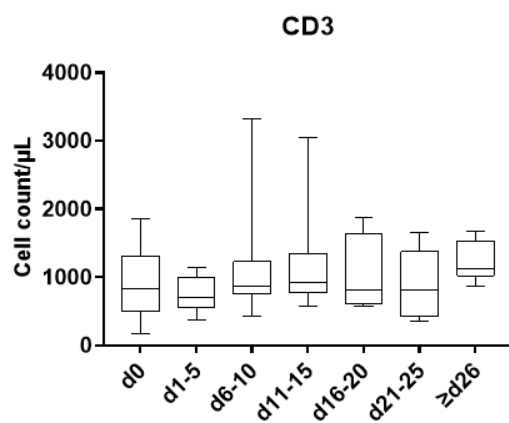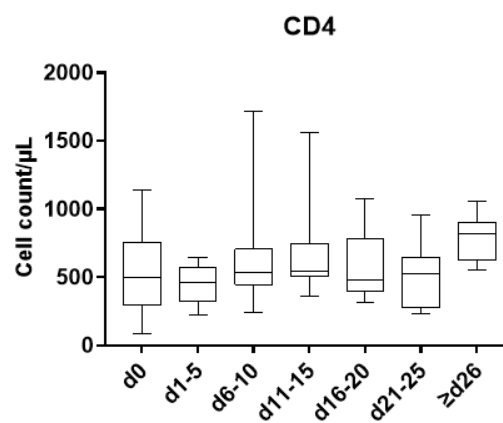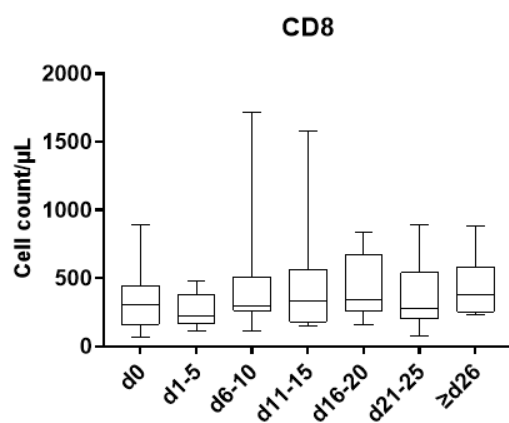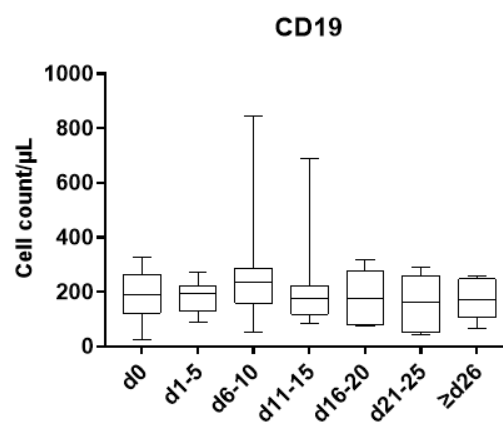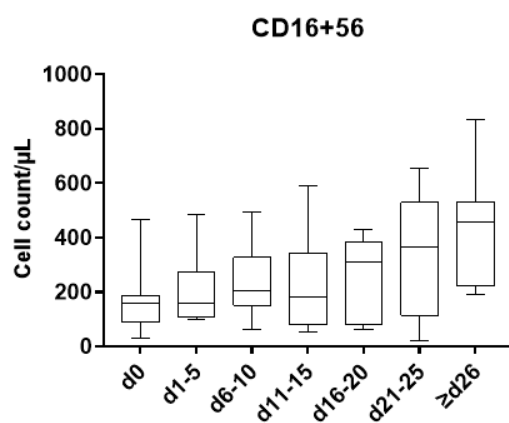

Supplement: FIG S2 [file mSphere.00922-20-sf002.pdf]

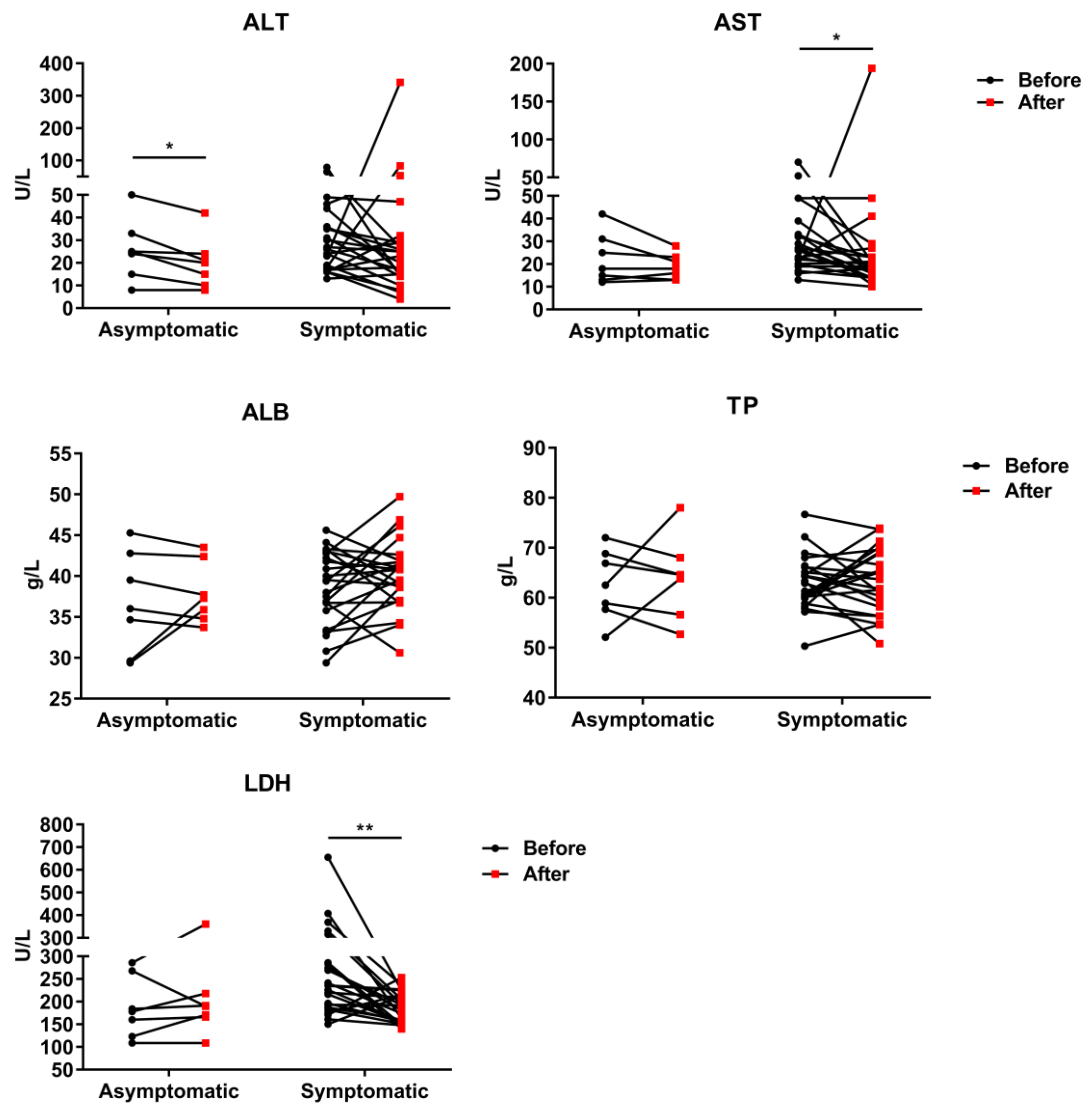

Supplement: FIG S3 [file mSphere.00922-20-sf003.pdf]
