# Supplementary material for: Descriptive, Retrospective Study of the Clinical Characteristics of Asymptomatic COVID-19 Patients
Source: mSphere. 2020 Oct 7;5(5):e00922-20. doi: 10.1128/mSphere.00922-20 (PMC7568656; doi:10.1128/mSphere.00922-20)
Supplement: TABLE S1 [file mSphere.00922-20-st001.pdf]

Supplementary Table 1. Selected markers of symptomatic and asymptomatic COVID-19 patients before and after treatment

Markers	Symptomatic				Asymptomatic			
	**Before (n=26)**	**After (n=26)**	z	P value	**Before (n=12)**	**After (n=12)**	z	P value
LYM	1.27(0.90,1.85)	1.81(1.32,2.13)	2.693	**0.007**	1.06(0.82,1.70)	1.13(0.90,1.92)	0.784	0.433
EOS	0.03(0.02,0.10)	0.14(0.07,0.19)	3.369	**0.001**	0.09(0.04,0.18)	0.09(0.06,0.21)	0.134	0.894
BASO	0.02(0.01,0.03)	0.04(0.02,0.05)	2.902	**0.004**	0.03(0.01,0.03）	0.03(0.02,0.04)	1.292	0.196
	**Before (n=23)**	**After (n=23)**			**Before (n=7)**	**After (n=7)**		
ALT	26.00(17.00,36.00)	23.00(14.00,29.00)	1.552	0.121	25.00(15.00,33.00)	20.00(10.00,24.00)	2.201	**0.028**
AST	26.00(20.00,33.00)	19.00(14.00,23.00)	2.417	**0.016**	18.00(13.00,31.00)	18.00(13.00,23.00)	1.156	0.248
TP	62.90(59.90,66.10)	63.70(58.20,69.10)	0.228	0.820	62.50(57.70,68.80)	64.60(56.60,68.00)	0.169	0.866
ALB	39.40(35.80,42.80)	40.80(37.00,41.90)	1.399	0.162	36.00(29.60,42.80)	37.30(34.80,42.40)	0.169	0.866
LDH	235.00(187.00,286.00)	192.00(156.00,215.00)	3.148	**0.002**	178.00(123.00,268.00)	190.00(166.00,218.00)	0.943	0.345
	**Before (n=18)**	**After (n=18)**						
$CD3^+$	821.50（589.50,1317.50）	1074.50(846.25,1628.50)	1.982	**0.048**				
$CD4^+$	458.50(349.25,760.25)	642.00(478.00,790.75)	1.198	0.231				
$CD8^+$	309.50(196.00,457.25)	378.50(269.75,636.50)	2.33	**0.020**				
$CD19^+$	197.00(154.25,299.25)	221.50(136.25,260.00)	0.741	0.459				
$CD16^+56^+$	160.50(88.50,210.00)	246.50(100.75,359.75)	3.027	**0.002**				
